# Macranthoidin B restrains the epithelial-mesenchymal transition through COX-2/PGE_2_ pathway in endometriosis

**DOI:** 10.3389/fphar.2024.1492098

**Published:** 2024-12-12

**Authors:** Yi Ding, Xiaoqian Yang, Qinghua Wei, Xuanming Bi, Yuxin Zhang, Yuxia Ma, Meisen Yang, Xiaoyu Xu, Cong Li, Qin Wang, Yi Chen

**Affiliations:** ^1^ School of Chinese Materia Medica, Chongqing University of Chinese Medicine, Chongqing, China; ^2^ College of Pharmaceutical Sciences and Chinese Medicine, Southwest University, Chongqing, China; ^3^ Traditional Chinese Medicine Industry Center of Xiushan Tujia & Miao Autonomous County, Agriculture and Rural Affairs Committee of Xiushan Tujia & Miao Autonomous County, Chongqing, China; ^4^ Department of Obstetrics and Gynecology, First Affiliated Hospital of Chongqing Medical University, Chongqing, China; ^5^ Department of Pharmacy, Chongqing Hospital of Traditional Chinese Medicine, Chongqing, China

**Keywords:** macranthoidin B, endometriosis, COX-2/PGE2 pathway, epithelial-mesenchymal transition, invasion and metastasis

## Abstract

**Introduction:**

Macranthoidin B is one of the primary and unique triterpenoid saponin metabolites from *Lonicera macranthoides* Hand. –Mazz, which is used to treat endometriosis (EMS) in traditional Chinese medicine. However, the effect of macranthoidin B remains unknown in EMS. This study aimed to elucidate the effect and mechanism of macranthoidin B in EMS.

**Methods:**

Using rat autograft EMS model, the volume of ectopic endothelium, the histopathology, serum E_2_ and PROG were evaluated after macranthoidin B’s treatment. In primary endometriotic stromal and HEC1-B cells, the invasion and metastasis were assessed by scratch wound and Transwell tests. The epithelial-mesenchymal transition and COX-2/PGE_2_ pathway were examined *in vivo* and *in vitro*. Macranthoidin B were combined with LPS or celecoxib.

**Results:**

In a rat autograft EMS model, macranthoidin B suppressed ectopic lesion volume, improved histopathological morphology, and regulated serum estradiol (E2) and progesterone (PROG) levels. Additionally, macranthoidin B inhibited invasion and metastasis of primary endometriotic stromal cells and HEC1-B cells. Mechanistically, macranthoidin B suppressed COX-2/PGE_2_ pathway and epithelial-mesenchymal transition both *in vivo* and *in vitro*. LPS, the COX-2/PGE2 pathway activator, showed the promotion of epithelial-mesenchymal transition, invasion and metastasis. Macranthoidin B exhibited the antagonistic effects against LPS. Celecoxib, the COX-2/PGE2 pathway inhibitor, restrained the epithelial-mesenchymal transition, invasion and metastasis. This effect of celecoxib was enhanced by macranthoidin B.

**Discussion:**

Macranthoidin B prevents epithelial-mesenchymal transition through COX-2/PGE2 pathway in EMS. It will facilitate the macranthoidin B’s development and broaden its potential application.

## Graphical Abstract

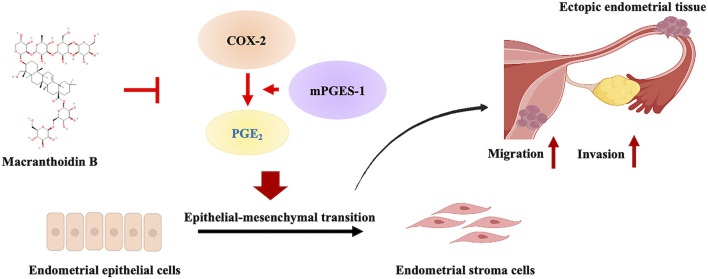

## 1 Introduction

In traditional Chinese medicine, *Lonicera macranthoides* Hand. –Mazz shows its anti-inflammatory properties in the clinical treatment of EMS (Liu and Shi, 2020; Chen and Xu, 2022). Previously, both its extract and its saponin metabolites downregulate COX-2 and PGE_2_ in inflammatory models and macrophage cells (Guan et al., 2014; Zeng et al., 2020a). Macranthoidin B, one of the main triterpenoid saponin metabolites, exhibits limited activities in cancer, such as the proliferative reduction, apoptosis and oxidant stress enhancements (Fan et al., 2018; Tan et al., 2023). However, the effect and mechanism of macranthoidin B had not been clarified in EMS.

EMS is an estrogen-dependent inflammatory disorder of the endometrium that is characterized by the presence of functionally active endometrial tissue growing outside the uterus. High E_2_ and dysregulated PROG productions are the consistently observed endocrine feature of EMS (Chantalat et al., 2020; MacLean and Hayashi, 2022). EMS is associated with the increase in the risk of epithelial ovarian cancer (Vercellini et al., 2014). Now, nonsteroidal anti-inflammatory drugs (NSAIDs) and hormonal therapies are the main therapeutic options in clinic (Taylor et al., 2021a). As a prevalent gynecological disorder, the activation of epithelial-mesenchymal transition promotes invasion and metastasis in EMS (Zhang C. et al., 2021). The process of epithelial-mesenchymal transition is characterized by a decrease in E-cadherin and an increase in Vimentin, N-cadherin, Twist, Snail, Slug, and Zeb1/2, which allows cells to invade and metastasize (Debnath et al., 2022). As a common gynecological inflammatory disease, EMS is accompanied with COX-2/PGE_2_ pathway upregulation (Taylor et al., 2021a). In recent years, COX-2/PGE_2_ pathway has been implicated in promoting epithelial-mesenchymal transition of tumors (Zhang H. et al., 2021; Zhao et al., 2024). However, it is unknown whether COX-2/PGE_2_ pathway regulates epithelial-mesenchymal transition in EMS.

This study aimed to determine the therapeutic effects of macranthoidin B on EMS in rat EMS model and endometrial cells. The impacts of macranthoidin B on COX-2/PGE_2_ pathway, epithelial-mesenchymal transition, invasion and metastasis were investigated both *in vivo* and *in vitro*. Additionally, the effects of macranthoidin B, combined with a COX-2/PGE_2_ pathway activator or inhibitor, were assessed separately in epithelial-mesenchymal transition, invasion and metastasis.

## 2 Methods

### 2.1 Chemicals

**FIGURE 1:**
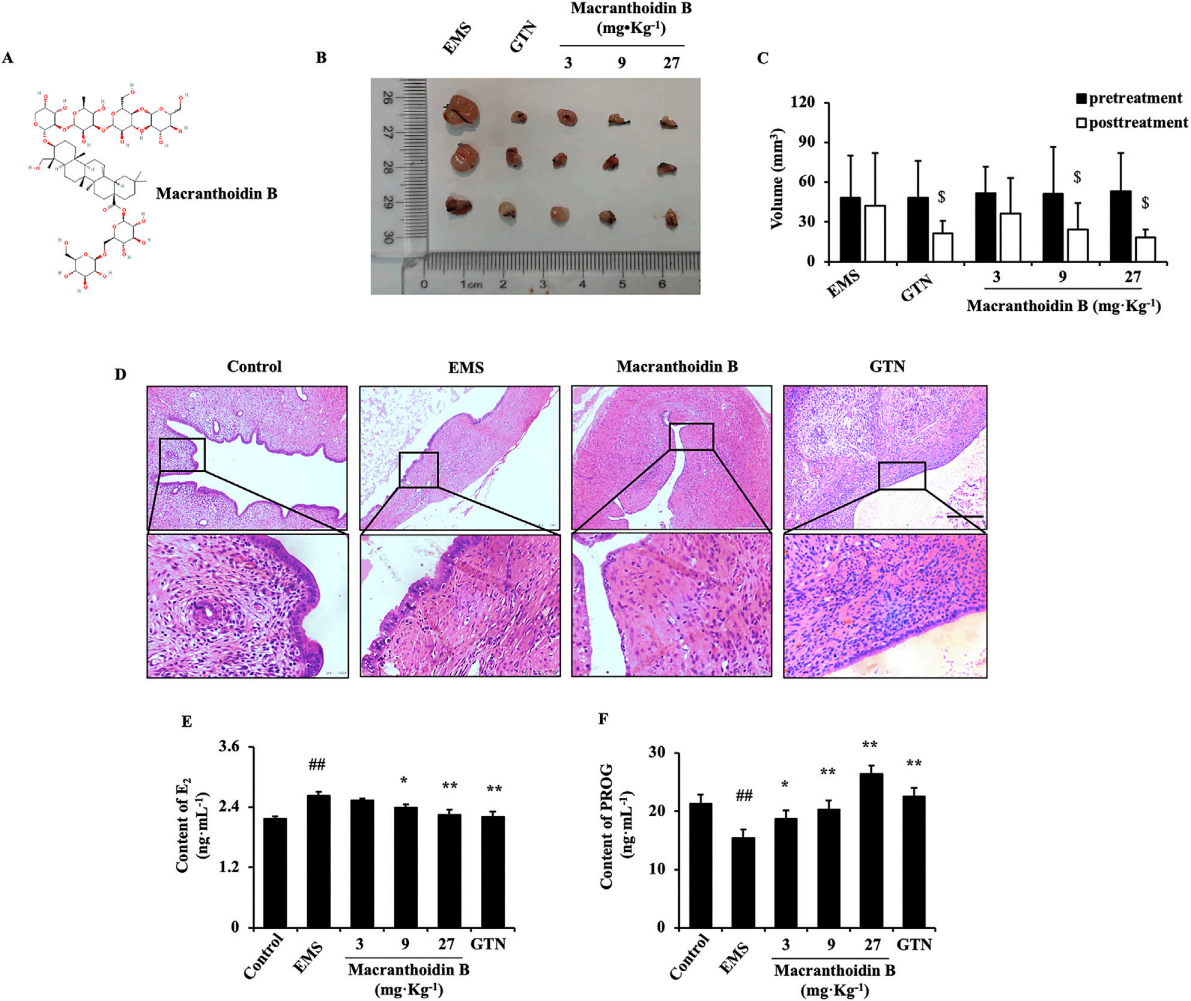
Macranthoidin B inhibited the growth of endometriotic tissues *in vivo* **(A)** The chemical structure of macranthoidin B **(B, C)** The volume of isolated ectopic lesions were calculated by vernier caliper after 28 days treatment. **(D)** Using H&E staining, pathological changes were observed. **(E, F)** E_2_ and PROG levels in serum were investigated by ELISA assay. $*P*< 0.05 to pretreatment, #*P*< 0.05 to control, ##*P*< 0.01 to control, *P< 0.05 to EMS, **P< 0.01 to EMS. Columns, mean (n = 5). Bars, SD. Scale bar = 100 μm. EMS, endometriosis; GTN, gestrinone.

Macranthoidin B (20200822, purity ≥98%, Nanjing Spring and Autumn Biological Engineering Co., Ltd., China) was dissolved separately in physiological saline or PBS for rat administration or endometrial cells treatment (Figure 1A). LPS (BS904, purity ≥98%, Sigma-Aldrich, United States) or celecoxib (EA9450, Pfizer Inc., United States) was dissolved in PBS or DMSO, respectively. Gestrinone was selected as a standard (53200201, Zizhu Pharmaceutical Co., Ltd., Beijing, China).

### 2.2 Rat autograft EMS model

Female SD rats, weighing 180–220 g and aged 6–7 weeks, were purchased from Hunan Slake Jingda Experimental Animal Co., Ltd. [Certification No: SCXK (Xiang) 2019-0004]. The rats were housed at a temperature of 20°C ± 2°C with a 12-h light/dark cycle and had free access to food and water at the Experimental Center, College of Pharmaceutical Sciences, Southwest University. The experimental protocol was approved by the Experimental Animal Ethics Review Committee of Southwest University (Approval No. IACUC-20210130-04), ensuring compliance with anesthesia and humane methods to minimize suffering.

The molding method and criteria of autograft EMS model were established according to the previous protocols (Dai et al., 2023). In summary, the left uterus of female rats was ligated, excised, and placed in a saline dish. It was then cut into approximately 5 mm^2^ endometrial segments, which were sutured to the right abdominal wall. After 28 days, the volume of ectopic tissue was measured by a vernier caliper with the formula (0.52 × length × width × height). The autograft section grew very well, for example, volume >8 mm^3^, blood vessels, and inflammatory encapsulation. It indicated the successfully established model. 30 female SD rats were operated for EMS establishment. The successful 25 EMS rats were randomly divided into EMS, 3, 9, 27 mg kg^−1^ macranthoidin B, and 0.5 mg kg^−1^ gestrinone groups. There were 5 rats per group. There were no significant differences in endometriotic volume among the groups before treatment. Another 5 normal female rats were treated as control group. Physiological saline was administered in control and EMS groups. All above groups were administered consecutive 28 days by gavage.

### 2.3 Primary endometriotic stromal cells and HEC1-B cells culture

For primary endometriotic stromal cells (ESCs), methods of cell acquisition and culture followed those outlined in a previous study (Dai et al., 2023). The study was approved by the First Affiliated Hospital of Chongqing Medical University (Permission 2019-059). Briefly, fresh ovarian EMS tissues were minced and digested by 0.1% collagenase I (Sigma-Aldrich, United States) for 90 min at 37°C. After separated by a 400 screen mesh, primary ESCs were cultured within DMEM/F12 medium (Gibco, NY, United States) supplemented with 10% FBS (Hyclone, Logan, United States) at 37°C with 5% CO_2_.

The endometrial cancer cell line, named HEC1-B, was purchased from Chinese Type Culture Collections (Wuhan, China). HEC1-B cells were cultured in MEM medium (Gibco, Grand Island, NY, United States) with 10% FBS (Hyclone, Logan, United States) at 37°C with 5% CO_2_.

### 2.4 Hematoxylin and eosin (H&E) staining, ELISA assay and immunofluorescence staining

For H&E staining, eutopic endometrium and ectopic endometrium were collected and fixed in paraformaldehyde in the end of administration. Then sections were stained and photographed under a microscope (DFC310 FX, Leica, Germany).

For ELISA assay, E_2_ and PROG detection in the serum proceed according to the published paper (Zhang C. et al., 2021). The PGE_2_ levels of ectopic endometrial tissue or cell supernatant were examined by rat or human PGE_2_ ELISA kits. All kits were used according to the instructions (SinoBestBio, Shanghai, China).

For immunofluorescence staining, rabbit anti-E-cadherin or anti-Vimentin antibodies (1:200 dilution; Proteintech, China) were applied to the endometrial tissue. Afterward, the tissues were treated with Alexa Fluor 594 or FITC-labeled goat anti-rabbit IgG secondary antibody (1:100 dilution; Beyotime, China). The sections were then counterstained with DAPI (Beyotime, China) and analyzed under a Leica microscope (Germany).

### 2.5 Cell viability assay

The previously reported method was employed for cell viability assay (Dai et al., 2023). Briefly, cells in 96-well plate were treated with macranthoidin B for 24 h. Then, MTT was used for incubation, and a microplate reader was performed for measurement. The 24 h IC_50_ values were calculated by GraphPad Prism Software (7.04 version, United States).

### 2.6 Scratch wound and transwell assays

The scratch wound and Transwell assays were conducted following the previous methodology (Zhang C. et al., 2021). For scratch wound assay, 6-9×10^4^ cells were cultured in 24-well plate until the 80% density. After being scratched by 1 mL pipette tip, cells were treated with macranthoidin B and observed under a microscope. Migration rate = (average scratch area at 0 h - average scratch area at 24 h)/average scratch area at 0 h × 100 %.

For the Transwell assay, the upper chamber included 3-5×10^4^ endometrial cells and serum-free medium in matrigel-coated Transwell inserts (Corning, New York, United States), while 10% FBS medium was added to the lower chamber. After 24 h, cell numbers in the bottom were fixed and counted in 3-5 random fields at ×100 magnification.

### 2.7 RT-qPCR

RNA isolation and cDNA synthesis were adhered to the previous approach (Zhang C. et al., 2021). SYBR™ Green Master Mix (Thermo Fisher, Waltham, United States) was used for gene expression with 2^−ΔΔCT^ calculation. Primer sequences of mRNA were produced by Shenggong Biotechnology (Shanghai, China) (Table 1).

**TABLE 1:** Primer sequences for RT-qPCR.

Species	Primer name	Sequences (5′–3′)
Human	*CDH1-F*	CTT​ACA​CCA​TCC​TCA​GCC​AAG​A
	*CDH1-R*	GCC​TCA​AAA​TCC​AAG​CCC​GTG
	*CDH2-F*	TTC​CAT​CCT​GCG​TGT​GAA​GG
	*CDH2-R*	GGC​TCA​AGT​CAT​AGT​CCT​GGT
	*VIM-F*	GGC​GAG​GAG​AGC​AGG​ATT​T
	*VIM-R*	GGG​TAT​CAA​CCA​GAG​GGA​GTG​A
	*SNAIL-F*	CCT​GTC​TGC​GTG​GGT​TTT​TG
	*SNAIL-R*	ACC​TGG​GGG​TGG​ATT​ATT​GC
	*TWIST-F*	TCT​CGG​TCT​GGA​GGA​TGG​A
	*TWIST-R*	CTG​CCC​GTC​TGG​GAA​TCA​CT
	*ZEB2-F*	ACT​CCT​GTC​TGT​CTC​GCA​A
	*ZEB2-R*	GAT​GTG​GTC​TTT​TTC​CTG​TGT​GT
	*PTGS2-F*	ATC​CCC​TTC​TGC​CTG​ACA​CC
	*PTGS2-R*	ATT​CCT​ACC​ACC​AGC​AAC​CCT​G
	*PTGES-F*	ACC​AGC​CAC​TCA​AAG​GAA​CT
	*PTGES-R*	CAC​ACA​TCT​CAG​GTC​ACG​GG
	*GAPDH-F*	AAT​GGG​CAG​CCG​TTA​GGA​AA
	*GAPDH-R*	GCC​CAA​TAC​GAC​CAA​ATC​AGA​G
Rat	*Cdh1-F*	CTG​GAC​CGA​GAG​AGT​TAC​CC
	*Cdh1-R*	GGC​ACC​GAC​CTC​ATT​CTC​AA
	*Cdh2-F*	GCT​TCT​GGC​GGC​CTT​GCT​TCA
	*Cdh2-R*	GCG​TAC​ACT​GTG​CCG​TCC​TCA​TCC
	*Vim-F*	CCT​TGA​CAT​TGA​GAT​TGC​CA
	*Vim-R*	GTA​TCA​ACC​AGA​GGG​AGT​GA
	*Snail-F*	CTG​CTT​GGC​TCT​CTT​GGT​GG
	*Snail-R*	AGG​AGG​GAT​GGG​ACT​ATT​GC
	*Slug-F*	ACA​TTA​GAA​CAC​ACA​CTG​GGG​A
	*Slug-R*	CTG​GAG​AAG​GTT​TTG​GAG​CAG
	*Zeb1-F*	GATGGGGCTGCGGATGAG
	*Zeb1-R*	GCA​GGG​TGC​TCT​GGG​TCA​TA
	*Zeb2-F*	CTC​CGA​TTC​CTG​TCT​GTC​TCG
	*Zeb2-R*	TGT​GGT​CTC​TTT​CCT​GTG​TGT
	*Twist-F*	ACC​CTC​ACA​CCT​CTG​CAT​TC
	*Twist-R*	CAG​TTT​GAT​CCC​AGC​GTT​TT
	*Ptgs2-F*	ACG​GAC​TTG​CTC​ACT​TTG​TTG
	*Ptgs2-R*	GCA​GCC​ATT​TCT​TTC​TCT​CCT​GT
	*Ptges-F*	CCC​TGT​CCC​CTG​TTG​ATA​CC
	*Ptges-R*	TGC​GGT​TTT​TAG​CGG​TTG​G
	*Gapdh-F*	AGA​CAG​CCG​CAT​CTT​CTT​GT
	*Gapdh-R*	CTT​GCC​GTG​GGT​AGA​GTC​AT

### 2.8 Western blotting

Briefly, the protein of cells and tissues were lysed and extracted by RIPA (Dingguo Changsheng, China). Then the proteins were separated by SDS-PAGE, and transferred to PVDF membrane (Millipore, Billerica, United States). Proteins on the membrane were bound with the primary antibodies and HRP-labeled goat anti-rabbit secondary antibody (Table 2). The membranes were reacted with electrochemiluminescence (ECL) enhanced Western blotting Substrate (Bioground Biotechnology Co, Ltd., China) for chemiluminescence. Finally, the protein bands were visualized in Tanon 5200 imaging system (Tanon, China).

**TABLE 2:** Antibody information for Western blotting.

Antibody name	Dilution	Item number	Purchasing companies
rabbit anti-E-cadherin	1:1,000	20874-1-AP	Proteintech, China
rabbit anti-N-cadherin	1:1,000	22018-1-AP	Proteintech, China
rabbit anti-Vimentin	1:1,000	10366-1-AP	Proteintech, China
rabbit anti-COX-2	1:1,000	WL01750	Wanleibio, China
rabbit anti-β-actin	1:2000	81115-1-RR	Proteintech, China
HRP-labeled goat anti-rabbit	1:10,000	BS13278	Bioworld, China

### 2.9 Statistical analysis

All data were represented by mean ± SD. Two sets of data were compared with unpaired *t*-tests. The volume of ectopic endometrium pre- and post-treatment were compared with paired *t*-tests. Three or more sets of data were compared with one-way ANOVA. SPSS 26.0 statistical software was used for analysis. *P*< 0.05 indicated a significant difference.

## 3 Results

### 3.1 Macranthoidin B inhibited ectopic lesions in autograft EMS model

After continuous gavage for 28 days, the volume of ectopic endometrium was evaluated and compared with the pretreatment. Administration of 9 or 27 mg kg^−1^ macranthoidin B resulted in smaller transplants with less adhesion and fewer surface blood vessels. Gestrinone diminished the volume of ectopic endometrial tissues (Figures 1B, C). In H&E staining, the ectopic endometrium in EMS exhibited a similar structure to the normal endometrium in control. The cortex invagination formed more pseudo glands with abundant blood vessels and inflammatory cell infiltrations. Macranthoidin B or gestrinone ameliorated the ectopic pathological morphology, with fewer and smaller pseudo glands, fewer micro vascularity and inflammatory infiltrations (Figure 1D). In EMS, E_2_ in serum were remarkably enhanced, accompanied with the decrease of PROG. Macranthoidin B diminished E_2_ and raised PROG levels (Figures 1E, F). Notably, macranthoidin B had no influence on rat weight during the treatment (Supplementary Figure S1A).

### 3.2 Macranthoidin B prohibited epithelial-mesenchymal transition and COX-2/PGE_2_ pathway *in vivo*

**FIGURE 2:**
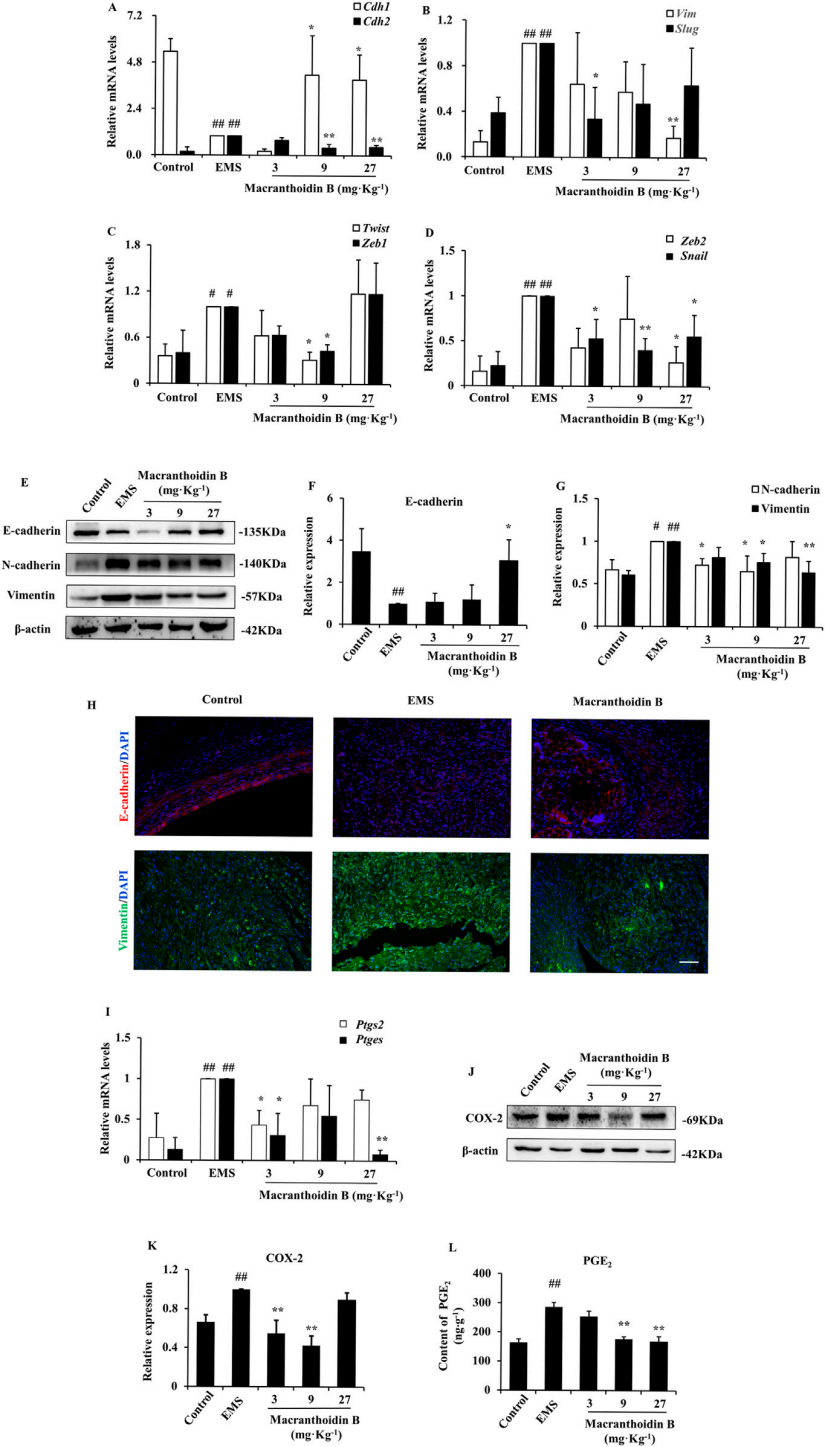
Macranthoidin B downregulated epithelial-mesenchymal transition and COX-2/PGE_2_ pathway in endometriotic tissues. **(A–D)** The mRNA levels of *Cdh1*, *Cdh2*, *Vim*, *Twist*, *Slug*, *Snail*, and *Zeb1/2* were measured by RT-qPCR. **(E–G)** The protein levels of E-cadherin, N-cadherin, and Vimentin were detected by Western blotting assay. **(H)** Detection of E-caderin and Vimentin in ectopic tissue by immunofluorescence assay. **(I)** The gene expression of *Ptgs2* and *Ptges* were tested by RT-qPCR. **(J–L)** The contents of COX-2 and PGE_2_ were analyzed by Western blotting and ELISA assays. #*P*< 0.05 to control, ##*P*< 0.01 to control, *P< 0.05 to EMS **P< 0.01 to EMS. Columns, mean (n = 3). Bars, SD. Scale bar = 100 μm. EMS, endometriosis.

The expression of the epithelial gene *Cdh1* was low in the EMS group, while the mesenchymal genes were increased, such as *Cdh2*, *Vim*, *Twist*, *Slug*, *Snail*, and *Zeb1/2*. Macranthoidin B obviously reversed the change of gene expression (Figures 2A–D). Furthermore, the E-cadherin protein was remarkably reduced in the EMS group, while N-cadherin and Vimentin proteins were increased. Macranthoidin B significantly promoted the protein levels of E-cadherin, accompanied by downregulation of N-cadherin and Vimentin (Figures 2E–G). In immunofluorescence assay, the downregulation of E-cadherin and the upregulation of Vimentin displayed in the ectopic tissues of EMS. E-cadherin was subsequently restored following macranthoidin B treatment, accompanied with the inhibition of Vimentin (Figure 2H).

Similarly, the mRNA and protein levels of COX-2/PGE_2_ pathway were elevated in EMS compared with control. Using macranthoidin B, the mRNA levels of *PTGS2* and *PTGES* were obviously decreased (Figure 2I). This was accompanied by a reduction in COX-2 and PGE_2_ protein contents (Figures 2J–L).

### 3.3 Restriction of cell invasion and metastasis by macranthoidin B

**FIGURE 3:**
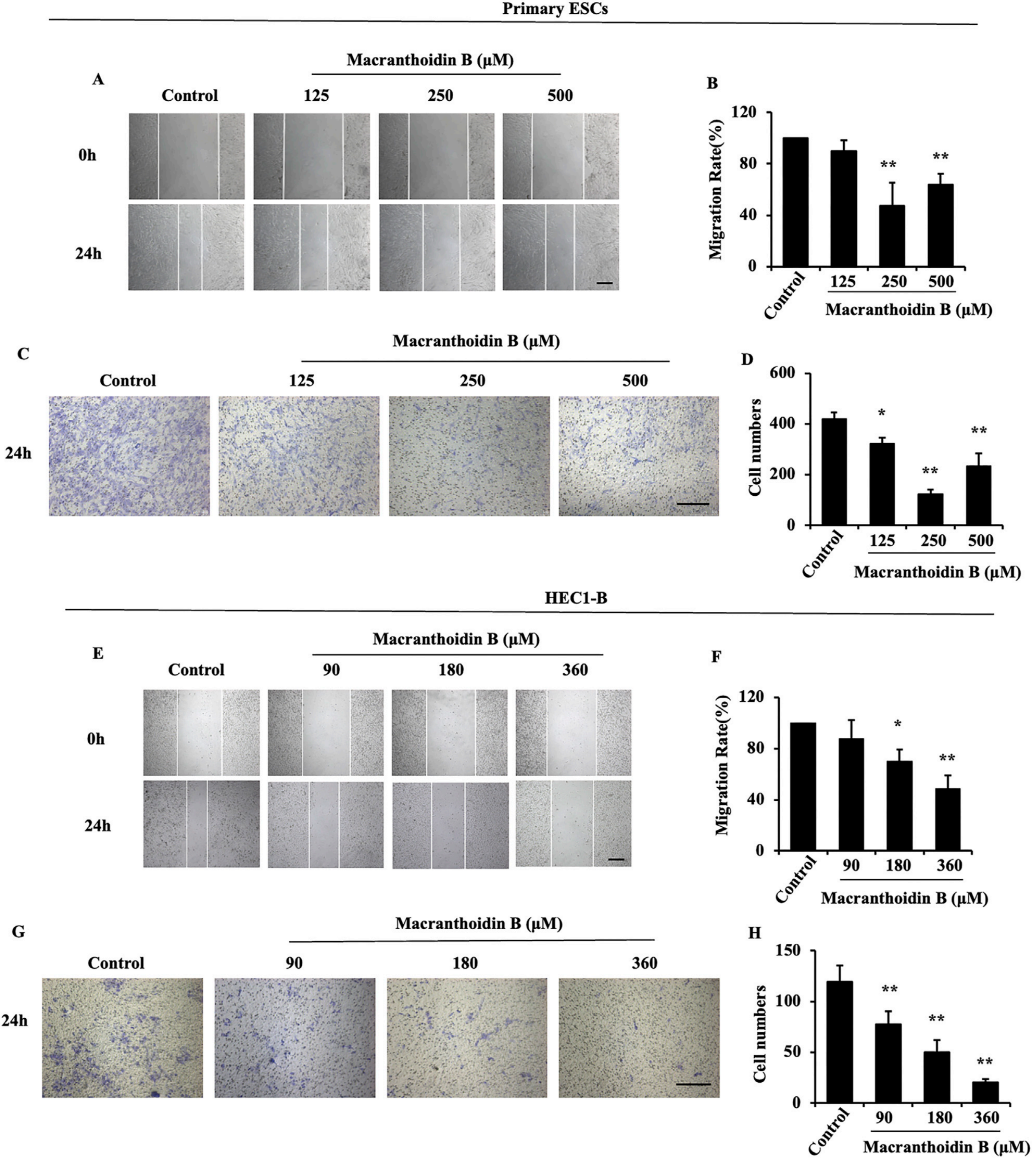
The suppression of invasion and metastasis by macranthoidin B in endometrial cells. **(A–D)** After being treated with macranthoidin B, the primary ESCs were tested in scratch wound and Transwell assays. **(E–H)** The cell migration and invasion ability of HEC1-B cells were observed in scratch wound and Transwell assays. *P< 0.05 to control, **P< 0.01 to control. Columns, mean (n = 3). Bars, SD. Scale bar = 100 μm. ESCs, endometriotic stromal cells.

According to the 24 h IC_50_, the primary ESCs or HEC1-B cells were treated with three concentrations of macranthoidin B (Supplementary Figures S1B, C). In primary ESCs, 2, 1, and 0.5 time of 24 h IC_50_ were performed. HEC1-B cells were treated with 1/2, 1/4, and 1/8 of 24 h IC_50_. After 24 h treatment, cells were prevented to cicatrize (Figures 3A, B, E, F). Migrating cells across insert membrane were significantly decreased in macranthoidin B groups vs. control group (Figures 3C, D, G, H). All above data implied that cell invasion and metastasis were resisted by macranthoidin B, particularly at 250 μM in primary ESCs, at 360 μM in HEC1-B cells.

### 3.4 Inhibition of epithelial-mesenchymal transition and COX-2/PGE_2_ pathway by macranthoidin B *in vitro*

**FIGURE 4:**
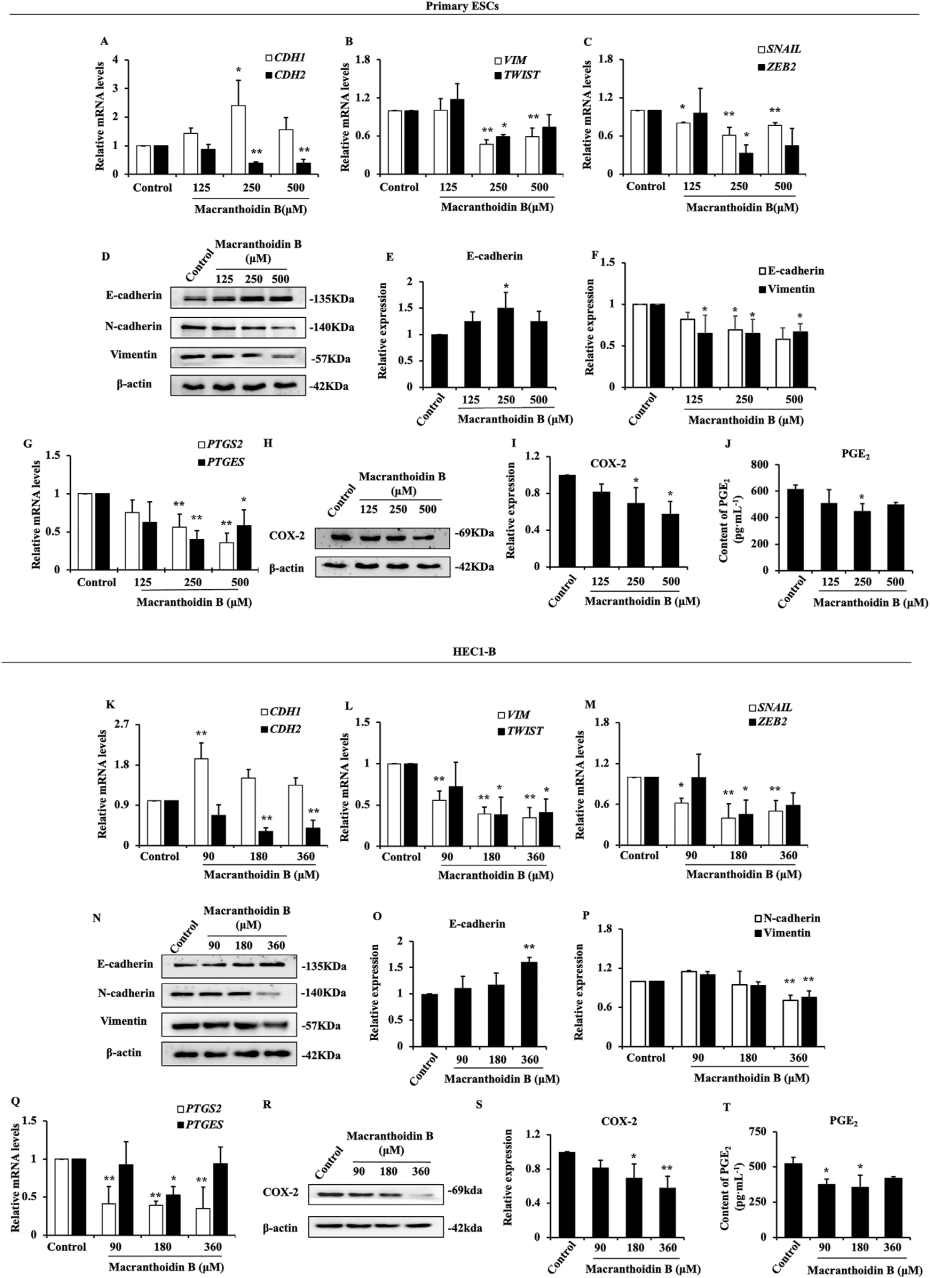
Macranthoidin B downregulated epithelial-mesenchymal transition and COX-2/PGE_2_ pathway *in vitro*. In primary ESCs **(A–C)** and HEC1-B cells **(K–M)**, the mRNA levels of *CDH1*, *CDH2*, *VIM*, *TWIST*, *SNAIL*, and *ZEB2* were measured by RT-qPCR. **(D–F, N–P)** The protein levels of epithelial-mesenchymal transition were tested by Western blotting assay. **(G, Q)** The gene expression of *PTGS2* and *PTGES* were investigated by RT-qPCR. **(H–J**, **R–T)** The content of COX-2 and PGE_2_ were analyzed by Western blotting and ELISA assays. *P< 0.05 to control, **P< 0.01 to control. Columns, mean (n = 3). Bars, SD. ESCs, endometriotic stromal cells.

In primary ESCs, *CDH1* gene expression was expanded at 250 μM macranthoidin B. The mRNA levels of *CDH2*, *VIM*, *TWIST*, *SNAIL*, and *ZEB2* were significantly reduced in macranthoidin B groups compared to control group (Figures 4A–C). Meanwhile, macranthoidin B exerted similar effect on HEC1-B cells (Figures 4K–M). Consequently, macranthoidin B increased the E-cadherin protein, and decreased the protein of mesenchymal markers, including N-cadherin and Vimentin in both types of endometrial cells (Figures 4D–F, N–P).

After using macranthoidin B for 24 h, there was a noticeable inhibitory effect on the gene expression of COX-2/PGE_2_ pathway in two endometrial cells (Figures 4G, Q). Subsequently, the levels of COX-2 and PGE_2_ proteins in endometrial cells were lessened in macranthoidin B groups compared with control group (Figures 4H–J, R–T).

### 3.5 Macranthoidin B restricted epithelial-mesenchymal transition induced by LPS

**FIGURE 5:**
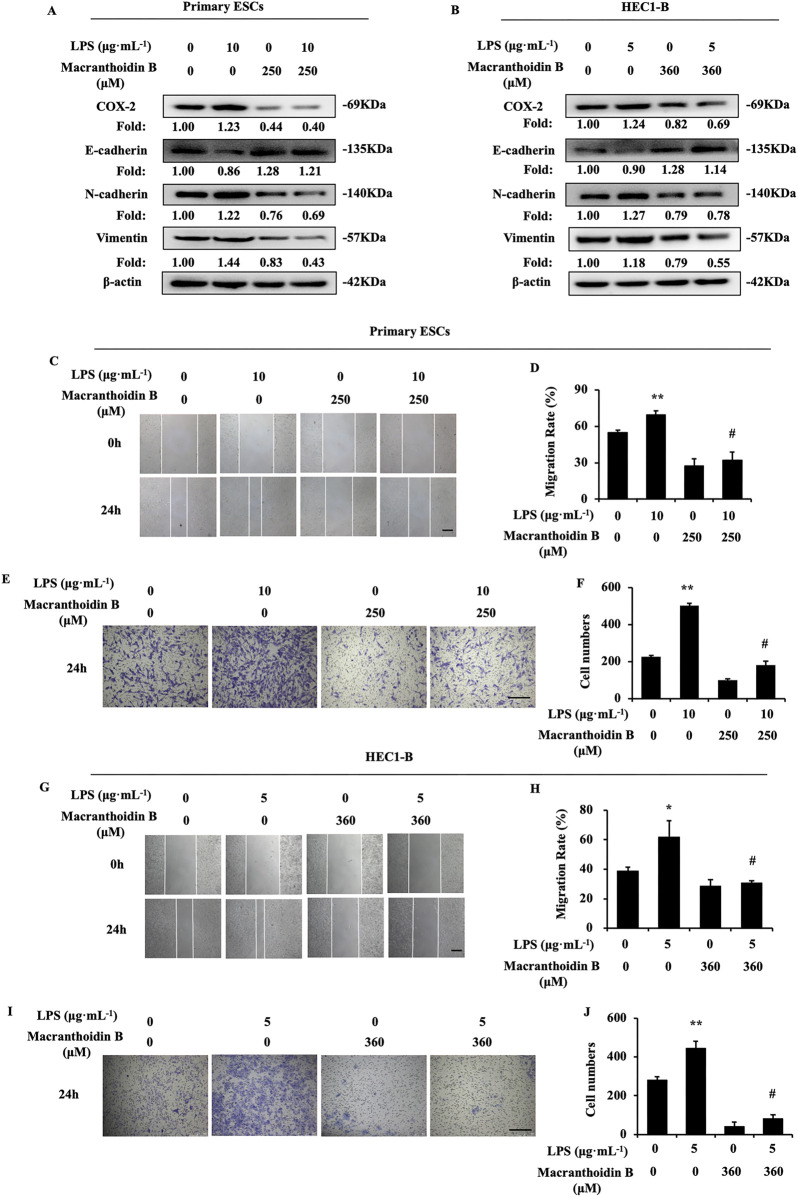
Macranthoidin B reversed LPS-induced activation of invasion and metastasis. **(A, B)** After using LPS or macranthoidin B, the expression of COX-2/PGE_2_ and epithelial-mesenchymal transition pathways were observed by Western blotting. **(C–J)** Cell migration and invasion ability were measured by scratch wound and Transwell assays in 24 h *P< 0.05 to control, **P< 0.01 to control. #*P*< 0.01 to LPS. Columns, mean (n = 3). Bars, SD. Scale bar = 100 μm. ESCs, endometriotic stromal cells.

LPS is a common inflammatory activator, which can promote COX-2, PTGES, and PGE_2_ (Alqinyah et al., 2023). In two types of endometrial cells, LPS stimulated the COX-2/PGE_2_ pathway, leading to epithelial mesenchymal transformation and enhancing invasive metastasis. Macranthoidin B reversed the LPS-activated COX-2/PGE_2_ pathway and epithelial-mesenchymal transition (Figures 5A, B). At the same time, invasion and metastasis were inhibited by macranthoidin B in scratching wound and Transwell assays (Figures 5C–J). Therefore, macranthoidin B exhibited the antagonistic effect on COX-2/PGE_2_ pathway, epithelial-mesenchymal transition, invasion and metastasis activated by LPS.

### 3.6 Synergism of macranthoidin B and celecoxib on epithelial-mesenchymal transition

**FIGURE 6:**
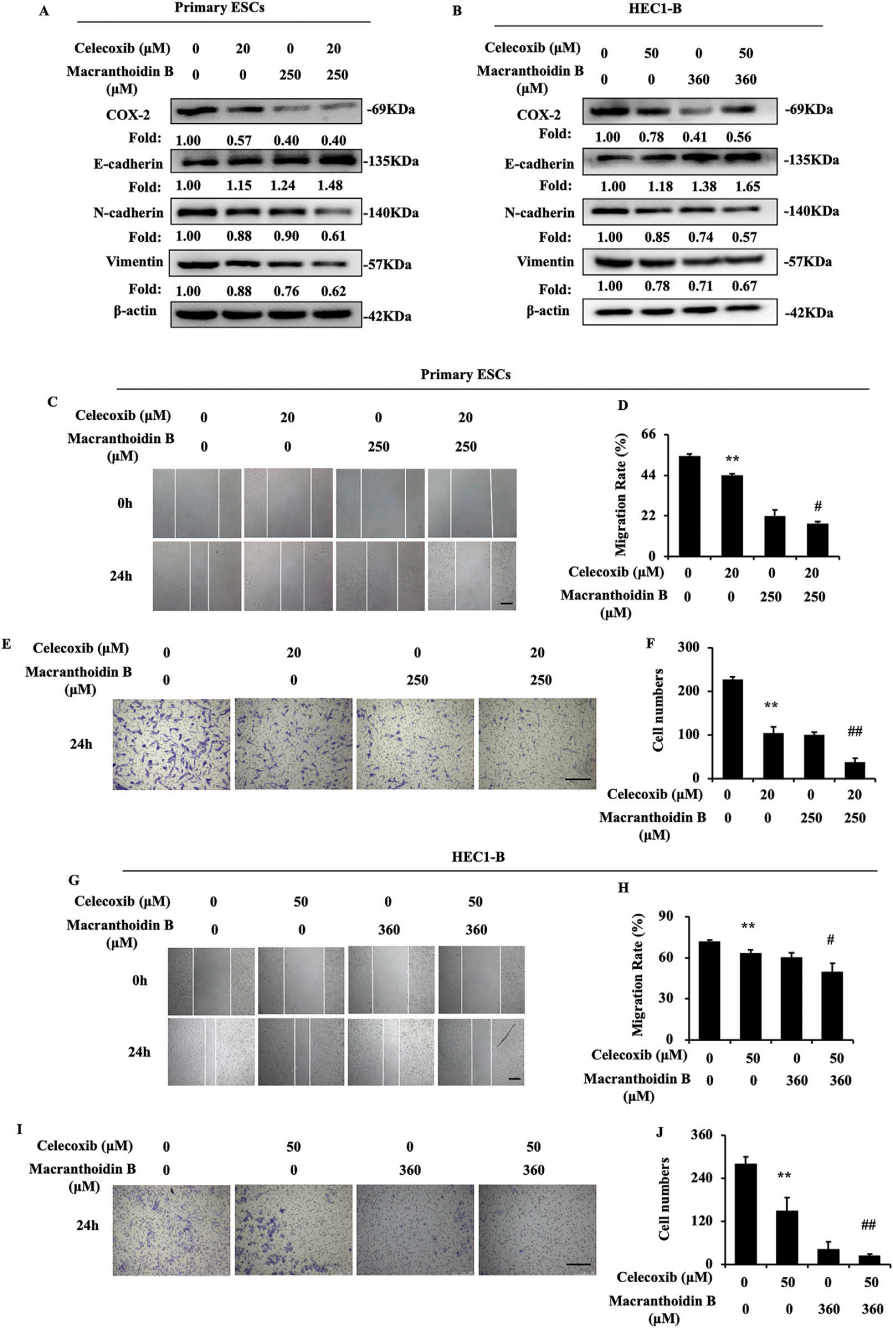
Macranthoidin B enhanced celecoxib-mediated attenuation of invasion and metastasis. **(A, B)** The protein levels of COX-2/PGE_2_ and epithelial-mesenchymal transition pathways were investigated by Western blotting after celecoxib or macranthoidin B treatment. **(C–J)** Cell migration and invasion ability were measured by scratch wound and Transwell assays in 24 h *P< 0.05 to control, **P< 0.01 to control. #*P*< 0.05 to celecoxib, ##*P*< 0.01 to celecoxib. Columns, mean (n = 3). Bars, SD. Scale bar = 100 μm. ESCs, endometriotic stromal cells.

Celecoxib is considered as a selective COX-2 inhibitor (Porat et al., 2023). In our study, celecoxib suppressed COX-2/PGE_2_ pathway and epithelial-mesenchymal transition in endometrial cells. Then, celecoxib restricted migration and invasion. Simultaneously, macranthoidin B not only enhanced celecoxib-mediated inhibition of COX-2/PGE_2_ pathway, but also augmented its effect on E-cadherin, N-cadherin, and Vimentin (Figures 6A, B). Cell migration and invasion were prevented by macranthoidin B and celecoxib (Figures 6C–J). In brief, macranthoidin B synergistically improved the celecoxib’s downregulation effect, especially in COX-2/PGE_2_ pathway and epithelial-mesenchymal transition.

## 4 Discussion

In this study, macranthoidin B significantly reduced the formation and ameliorated the pathological structure of ectopic endometrium. Moreover, macranthoidin B also decreased E_2_ and raised PROG levels. In addition, macranthoidin B hindered invasion and metastasis of endometrial cells. This was attributed to the blockade of epithelial-mesenchymal transition *via* COX-2/PGE_2_ pathway.

COX-2/PGE_2_ pathway is an important inflammatory pathway, in which COX-2 acts with mPGES-1 to mediate PGE_2_ synthesis (Akasaka and Ruan, 2016). COX-2/PGE_2_ pathway can influence the epithelial-mesenchymal transition in cancer. Activation of COX-2/PGE_2_ pathway causes the loss of the epithelial E-cadherin while it is boosting mesenchymal N-cadherin and Vimentin. Moreover, AGR2 and PGE_2_ receptor EP2 mediate the PGE_2_-induced epithelial-mesenchymal transition through regulating Snail (Cheng et al., 2014; Zhang H. et al., 2021). These changes facilitate the occurrence of epithelial-mesenchymal transition, thereby enhance the invasion and metastasis abilities (Che et al., 2017; Gómez-Valenzuela et al., 2021). This indicates a significant correlation between the COX-2/PGE_2_ pathway and epithelial-mesenchymal transition. EMS is a chronic inflammatory gynecological disease that affects many women of reproductive age. Clinical data indicate increasing COX-2/PGE_2_ expression in EMS patients (Cho et al., 2010; Peng et al., 2018). During EMS progression, the COX-2/PGE_2_ pathway indirectly promotes angiogenesis at ectopic sites (Jana et al., 2016). Recently, epithelial-mesenchymal transition has been proved to be the vital step in EMS progression (Zhang C. et al., 2021). Moreover, PGE_2_ also stimulates migration and epithelial-mesenchymal transition in normal endometrial epithelial cells (Kusama et al., 2021). Our investigation discovered overexpression of COX-2/PGE_2_ pathway and epithelial-mesenchymal transition in autograft EMS model. COX-2/PGE_2_ pathway was activated by LPS. Then epithelial-mesenchymal transition, invasion and metastasis were induced in endometrial cells. Conversely, using celecoxib, the downregulation of COX-2/PGE_2_ pathway led to the suppression of epithelial-mesenchymal transition, invasion and metastasis. However, further research is required to confirm the relationship in human EMS tissues or other EMS animal models.

*Lonicera macranthoides* Hand. –Mazz, also called *Lonicerae Flos*, has demonstrated pharmacological properties, such as antibacterial, antiviral, antipyretic, and anti-inflammatory activities (Li et al., 2020). Initially, *Lonicerae Flos* extract exhibits the anti-inflammatory action through the COX-2 reduction in the animal inflammation model and LPS-induced cellular model (Zeng et al., 2020a; Zeng et al., 2020a). The saponins are considered as the important material basis of anti-inflammatory effect. Previous studies has shown that both *Lonicerae Flos* and its saponins can suppress COX-2/PGE_2_ pathway (Guan et al., 2014; Zeng et al., 2020b). Lonimaranthoide VI, a saponin metabolite in *Lonicerae Flos*, also displays anti-inflammatory activity by regulating COX-2-induced PGE_2_ synthesis (Guan et al., 2014). Furthermore, macranthoidin B is one of the main triterpenoid saponin metabolites in *Lonicera macranthoides* Hand. –Mazz. Limited pharmacological studies indicate that it exhibits the anti-tumor effects (Fan et al., 2018; Tan et al., 2023). In our research, the effect of macranthoidin B on EMS was uncovered both *in vivo* and *in vitro*. Its mechanism involved the suppression of COX-2/PGE_2_ pathway, which subsequently caused the downregulation of epithelial-mesenchymal transition, invasion and metastasis. Notably, the dose levels of macranthoidin B lacked the dose response in primary ESCs. It might be related to the high selection of doses as a limitation. It is worthwhile to find more suitable doses and explore other mechanisms of macranthoidin B in EMS. Furthermore, paeonol, imperatorin, ginsenoside Rg3, ferulic Acid, ligustrazine, and tetrahydropalmatine also show the inhibition in EMS (Huang et al., 2020; Ma et al., 2021; Pang et al., 2021; Zhang C. et al., 2021). So it needs to screen more potential natural metabolites in the future.

In addition, EMS is an estrogen-dependent disease (Marquardt et al., 2019). E_2_ secretion in EMS is higher, despite of lower PROG secretion (Patel et al., 2017; Marquardt et al., 2019). Estrogen generation in ectopic endometrium can activate COX-2 and accelerate prostaglandin synthesis. This leads to a positive feedback loop, enhancing estrogen production and inflammation, which further promotes the EMS progression (Bulun et al., 2012; Takaoka et al., 2018). In accordance with these studies, we also detected the increasing E_2_ and decreasing PROG in EMS serum. The overexpression of COX-2/PGE_2_ pathway was found in ectopic endometrium. Macranthoidin B treatment resulted in the opposite changes of E_2_ and PROG, alongside the downregulation of COX-2/PGE_2_ pathway. Therefore, the mechanism of macranthoidin B can be further investigated in the regulation of the relationship between COX-2/PGE_2_ pathway and E_2_.

In summary, macranthoidin B showed the constraint on EMS progression. Macranthoidin B inhibited epithelial-mesenchymal transition *via* COX-2/PGE_2_ pathway. These findings provide a theoretical basis for the further development of macranthoidin B.

## Data Availability

The original contributions presented in the study are included in the article/Supplementary Material, further inquiries can be directed to the corresponding authors.

## Abbreviations

EMS, endometriosis; H&E, hematoxylin and eosin; MTT, 3-(4,5-dimethylthiazol-2-yl)-2–5-diphenyltetrazolium bromide; ELISA, enzyme linked immunosorbent assay; RT-qPCR, Real Time Quantitative PCR; LPS, lipopolysaccharides; DMSO, dimethyl sulphoxide; PBS, phosphate buffered saline; COX-2, cyclooxygenase-2; PGE_2_, Prostaglandin E synthase 2; mPGES-1, microsomal prostaglandin E synthases 1; E_2_, estradiol; PROG, progestogen; ESCs, endometriotic stromal cells; ECL, electrochemiluminescence; NSAIDs, nonsteroidal anti-inflammatory drugs.

## References

[B1] Akasaka H. Ruan K.-H. (2016). Identification of the two-phase mechanism of arachidonic acid regulating inflammatory prostaglandin E2 biosynthesis by targeting COX-2 and mPGES-1. Archives Biochem. Biophysics 603, 29–37. 10.1016/j.abb.2016.04.011 27177970

[B2] Alqinyah M. Alhamed A. S. Alnefaie H. O. Algahtani M. M. Badr A. M. Albogami A. M. (2023). Targeting store-operated calcium entry regulates the inflammation-induced proliferation and migration of breast cancer cells. Biomedicines 11, 1637. 10.3390/biomedicines11061637 37371732 PMC10296208

[B3] Bulun S. E. Monsavais D. Pavone M. E. Dyson M. Xue Q. Attar E. (2012). Role of estrogen receptor-β in endometriosis. Seminars Reproductive Med. 30, 39–45. 10.1055/s-0031-1299596 PMC4034571 22271293

[B4] Chantalat E. Valera M. C. Vaysse C. Noirrit E. Rusidze M. Weyl A. (2020). Estrogen receptors and endometriosis. Int. J. Mol. Sci. 21, 2815. 10.3390/ijms21082815 32316608 PMC7215544

[B5] Che D. Zhang S. Jing Z. Shang L. Jin S. Liu F. (2017). Macrophages induce EMT to promote invasion of lung cancer cells through the IL-6-mediated COX-2/PGE2/β-catenin signalling pathway. Mol. Immunol. 90, 197–210. 10.1016/j.molimm.2017.06.018 28837884

[B6] Chen X. Xu C. (2022). Research progres on treatment of endometriosis from blood stasis, heat and toxin. Asia-Pac Trad. Med. 18 (09), 182–186. 10.19945/j.cnki.issn.1006-3250.2022.07.014

[B7] Cheng S. Y. Zhang H. Zhang M. Xia S. K. Bai X. M. Zhang L. (2014). Prostaglandin E₂ receptor EP2 mediates Snail expression in hepatocellular carcinoma cells. Oncol. Rep. 31, 2099–2106. 10.3892/or.2014.3074 24626807

[B8] Cho S. Park S. H. Choi Y. S. Seo S. K. Kim H. Y. Park K. H. (2010). Expression of cyclooxygenase-2 in eutopic endometrium and ovarian endometriotic tissue in women with severe endometriosis. Gynecol. Obstet. Invest 69, 93–100. 10.1159/000261017 20068324

[B9] Dai X. S. Wei Q. H. Guo X. Ding Y. Yang X. Q. Zhang Y. X. (2023). Ferulic acid, ligustrazine, and tetrahydropalmatine display the anti-proliferative effect in endometriosis through regulating Notch pathway. Life Sci. 328, 121921. 10.1016/j.lfs.2023.121921 37429417

[B10] Debnath P. Huirem R. S. Dutta P. Palchaudhuri S. (2022). Epithelial-mesenchymal transition and its transcription factors. Biosci. Rep. 42. 10.1042/BSR20211754 PMC8703024 34708244

[B11] Fan X. Rao J. Zhang Z. Li D. Cui W. Zhang J. (2018). Macranthoidin B modulates Key metabolic pathways to enhance ROS generation and induce cytotoxicity and apoptosis in colorectal cancer. Cell. Physiology Biochem. Int. J. Exp. Cell. Physiology, Biochem. Pharmacol. 46, 1317–1330. 10.1159/000489147 29689551

[B12] Gómez-Valenzuela F. Escobar E. Pérez-Tomás R. Montecinos V. P. (2021). The inflammatory profile of the tumor microenvironment, orchestrated by cyclooxygenase-2, promotes epithelial-mesenchymal transition. Front. Oncol. 11, 686792. 10.3389/fonc.2021.686792 34178680 PMC8222670

[B13] Guan F. Wang H. Shan Y. Chen Y. Wang M. Wang Q. (2014). Inhibition of COX-2 and PGE2 in LPS-stimulated RAW264.7 cells by lonimacranthoide VI, a chlorogenic acid ester saponin. Biomed. Rep. 2, 760–764. 10.3892/br.2014.314 25054024 PMC4106565

[B14] Huang R. Chen S. Zhao M. Li Z. Zhu L. (2020). Ginsenoside Rg3 attenuates endometriosis by inhibiting the viability of human ectopic endometrial stromal cells through the nuclear factor-kappaB signaling pathway. J. Gynecol. Obstet. Hum. Reprod. 49, 101642. 10.1016/j.jogoh.2019.101642 31563698

[B15] Jana S. Chatterjee K. Ray A. K. Dasmahapatra P. Swarnakar S. (2016). Regulation of matrix metalloproteinase-2 activity by COX-2-PGE2-pAKT Axis promotes angiogenesis in endometriosis. PLoS One 11, e0163540. 10.1371/journal.pone.0163540 27695098 PMC5047632

[B16] Kusama K. Fukushima Y. Yoshida K. Sakakibara H. Tsubata N. Yoshie M. (2021). Endometrial epithelial-mesenchymal transition (EMT) by menstruation-related inflammatory factors during hypoxia. Mol. Hum. Reprod. 27, gaab036. 10.1093/molehr/gaab036 33983443

[B17] Li Y. Li W. Fu C. Song Y. Fu Q. (2020). Lonicerae japonicae flos and Lonicerae flos: a systematic review of ethnopharmacology, phytochemistry and pharmacology. Phytochem. Rev. 19, 1–61. 10.1007/s11101-019-09655-7 32206048 PMC7088551

[B18] Liu J. Shi Y. (2020). Shi yanping uses traditional Chinese medicine to treat endometriosis from immune inflammation. J. Pract. Traditional Chin. Intern. Med. 34 (03), 38–41. 10.13729/j.issn.1671-7813.z20190673

[B19] Ma T. Liu P. Wei J. Zhao M. Yao X. Luo X. (2021). Imperatorin alleviated endometriosis by inhibiting the activation of PI3K/Akt/NF-κB pathway in rats. Life Sci. 274, 119291. 10.1016/j.lfs.2021.119291 33667515

[B20] Maclean J. A. Hayashi K. (2022). Progesterone actions and resistance in gynecological disorders. Cells 11, 647. 10.3390/cells11040647 35203298 PMC8870180

[B21] Marquardt R. M. Kim T. H. Shin J.-H. Jeong J.-W. (2019). Progesterone and estrogen signaling in the endometrium: what goes wrong in endometriosis? Int. J. Mol. Sci. 20, 3822. 10.3390/ijms20153822 31387263 PMC6695957

[B22] Pang C. Wu Z. Xu X. Yang W. Wang X. Qi Y. (2021). Paeonol alleviates migration and invasion of endometrial stromal cells by reducing HIF-1α-regulated autophagy in endometriosis. Front. Biosci. Landmark Ed. 26, 485–495. 10.52586/4961 34590461

[B23] Patel B. G. Rudnicki M. Yu J. Shu Y. Taylor R. N. (2017). Progesterone resistance in endometriosis: origins, consequences and interventions. Acta Obstetricia Gynecol. Scand. 96, 623–632. 10.1111/aogs.13156 28423456

[B24] Peng B. Zhan H. Alotaibi F. Alkusayer G. M. Bedaiwy M. A. Yong P. J. (2018). Nerve growth factor is associated with sexual pain in women with endometriosis. Reprod. Sci. 25, 540–549. 10.1177/1933719117716778 28673205

[B25] Porat D. Dukhno O. Partook-Maccabi M. Vainer E. Cvijic S. Dahan A. (2023). Selective COX-2 inhibitors after bariatric surgery: celecoxib, etoricoxib and etodolac post-bariatric solubility/dissolution and pharmacokinetics. Int. J. Pharm. 645, 123347. 10.1016/j.ijpharm.2023.123347 37633536

[B26] Takaoka O. Mori T. Ito F. Okimura H. Kataoka H. Tanaka Y. (2018). Daidzein-rich isoflavone aglycones inhibit cell growth and inflammation in endometriosis. J. Steroid Biochem. Mol. Biol. 181, 125–132. 10.1016/j.jsbmb.2018.04.004 29679753

[B27] Tan S. Liu Q. Yang J. Cai J. Yu M. Ji Y. (2023). Macranthoidin B (MB) promotes oxidative stress-induced inhibiting of hepa1-6 cell proliferation via selenoprotein. Biol. Trace Elem. Res. 201, 368–376. 10.1007/s12011-022-03120-x 35080709

[B28] Taylor H. S. Kotlyar A. M. Flores V. A. (2021a). Endometriosis is a chronic systemic disease: clinical challenges and novel innovations. Lancet London, Engl. 397, 839–852. 10.1016/S0140-6736(21)00389-5 33640070

[B29] Vercellini P. Vigano P. Somigliana E. Fedele L. (2014). Endometriosis: pathogenesis and treatment. Nat. Rev. Endocrinol. 10, 261–275. 10.1038/nrendo.2013.255 24366116

[B30] Zeng A. Hua H. Chen C. Liu L. Zhang M. Luo Y. (2020a). Comparative study on anti-inflammatory effect of Lonicerae japonicae flos and Lonicerae flos. China J. Chin. Materia Medica 16, 196–202. 10.19540/j.cnki.cjcmm.20200520.401 32893592

[B31] Zeng A. Hua Y. Chen C. Liu L. Zhang M. Luo Y. (2020b). Study on the anti-inflammatory pharmacological effects of *Lonicera japonica* and Lonicerae Flos. China J. Chin. Materia Medica 45, 3938–3944. 10.19540/j.cnki.cjcmm.20200520.401 32893592

[B32] Zhang C. Zhang Y. Pan H. Tan Y. Wei Q. Dai X. (2021a). Combination of ferulic acid, ligustrazine and tetrahydropalmatine attenuates epithelial-mesenchymal transformation *via* wnt/β-catenin pathway in endometriosis. Int. J. Biol. Sci. 17, 2449–2460. 10.7150/ijbs.60167 34326686 PMC8315018

[B33] Zhang H. Chi J. Hu J. Ji T. Luo Z. Zhou C. (2021b). Intracellular AGR2 transduces PGE2 stimuli to promote epithelial-mesenchymal transition and metastasis of colorectal cancer. Cancer Lett. 518, 180–195. 10.1016/j.canlet.2021.06.025 34216690

[B34] Zhao J. Zhu Q. Zhang Y. Li G. Zhang Y. Li F. (2024). Role of COX-2/PGE2/EP4 axis-induced macrophage functional activation in NSCLC development. Zhongguo Fei Ai Za Zhi 27, 245–256. 10.3779/j.issn.1009-3419.2024.101.05 38769827 PMC11110263

