# Supplementary material for: Macranthoidin B restrains the epithelial-mesenchymal transition through COX-2/PGE2 pathway in endometriosis
Source: Front Pharmacol. 2024 Dec 12;15:1492098. doi: 10.3389/fphar.2024.1492098 (PMC11669684; doi:10.3389/fphar.2024.1492098)
Supplement: Supplementary file 1 [file DataSheet1.pdf]

Figure S1

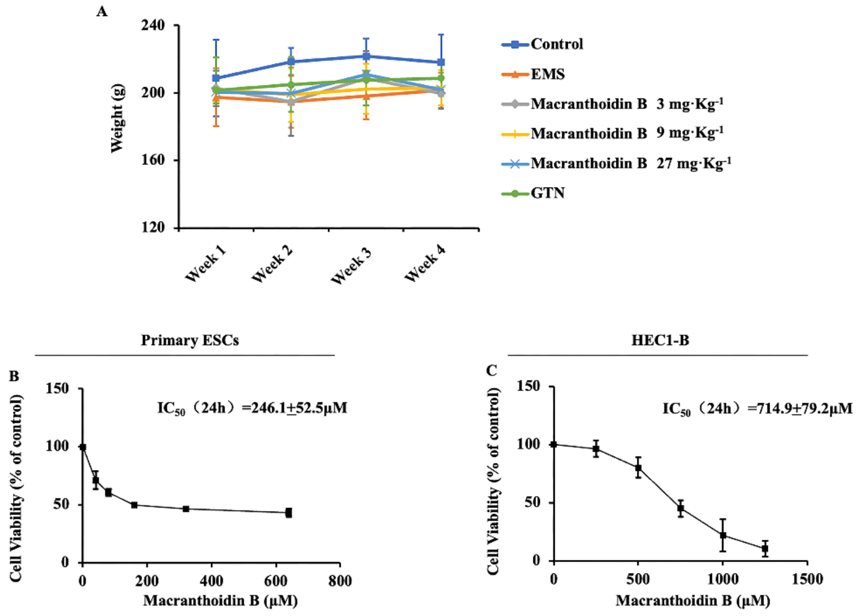

Figure S1. The effect of macranthoidin B on rat body weight and human endometrial cells. (A) Body weight was measured during the experimental period. **(B-C)** In primary ESCs and HEC1-B cells, 24h IC_{50} value of macranthoidin B were evaluated by MTT assay.
